# The Foreign Journals: Surgery

**Published:** 1839-10

**Authors:** 


### SURGERY.

*On the Treatment of Lateral Curvatures of the Spine, by the Subcutaneous Division of the Muscles of the Back and the Vertebral Column. Abstract of a Letter addressed to the Academy of Sciences.* By JULES GUERIN, M.D.

I HAVE the honour to communicate to the Academy the first result of a new operation, which I have already practised twelve times successfully in subjects affected with lateral curvature of the spine. This operation consists in the section of certain muscles of the back and vertebral column. The muscles which I have at present divided are the trapezius, the rhomboideus, the levator anguli scapulæ, the sacro-lumbalis, longissimus dorsi, and the semi-spinalis.

The greater number of lateral distortions of the spine are the result of active muscular contraction, and their anatomical varieties are the result of this contraction, variously exhibited in the muscles of the spine and back The active treatment of this class of deformities must, therefore, consist in the subcutaneous section of the muscles, to the shortening of which each deformity is due. The following are some details of the applications which I have made of this mode of treatment:

I have applied it to subjects of both sexes and of different ages: the youngest was thirteen, the oldest thirty-two years old. All the cases were deformed in the second or third degree, with proportionate twisting of the column, and gibbosity. In some a single division of the retracted muscles sufficed; in others, two or three had to be made. In all I obtained, immediately after the operation, a marked degree of straightening of the column; and in a young man of twenty-one, whose deformity had been subjected for eighteen months to mechanical treatment, I obtained, by dividing the corresponding longissimus dorsi and semi-spinalis, an immediate removal of the whole deviation. In other subjects I have been able to pursue, with a constant success, the treatment by mechanical means. In none of the twelve operations have I met with the least accident; there has been no hemorrhage; but little pain; no fever; and, in every case but one, immediate union of the wound has taken place, without suppuration. I may add that, though delicate, this operation may be performed almost as easily as the similar one in the neck or foot. *Gazette Médicale.* *June* 29, 1839

### *Statistics of Tracheotomy.*

Since the introduction of tracheotomy in croupal affections into France, there has, doubtless, been reason to deplore a great number of failures; it may be presumed, however, that one of the chief reasons of its insufficiency depends on the delay and the obstacles that are generally thrown in the way of its performance. The following are the results which the different most celebrated operators have by their own declarations, in a late discussion at the Royal Academy of Medicine in Paris, obtained.

| | Operations. | Cures. | Deaths. |
|---|---|---|---|
| M. Amussat . . . | 6 | 0 | 6 |
| Baudelocque . . . | 15 | 0 | 15 |
| Blandin . . . | 5 | 0 | 5 |
| Brettonneau . . . | 18 | 4 | 14 |
| Gerdy . . . | 6 | 4 | 2 |
| Roux . . . | 4 | 0 | 4 |
| Trousseau . . . | 80 | 20 | 60 |
| Velpeau . . . | 6 | 0 | 6 |
| | 140 | 28 | 112 |

So that, of 140 patients operated on, 28 only have been cured, and 112 have died.
*Journal des Connais. Méd. Juin,* 1839.

---

### *Gangrene resulting from the employment of a Starched Bandage in a case of Fracture of the Patella.* By Dr. Defer, of Metz.

A man, æt. 40, received a transverse fracture of the patella, from a fall. The medical officer who was called in, applied first a bandage, to bring the portions of bone together, and then a starched roller, which extended from the toes to the upper third of the thigh; the limb was then placed on an inclined plane. The patient was occasionally visited; but, as he suffered scarcely any pain, the apparatus was not altered. About six weeks after the accident his attendant, on proceeding to remove the apparatus, found the smell such as to induce the presumption that gangrene had supervened; and Dr. Defer was called in. He found the odour that exhaled from the limb such as could not for an instant permit a doubt of the existence of gangrene. The toes which the roller had not covered were mummified and completely insensible. On removing the bandage, the gangrene was seen to extend to within seven inches of the knee. The foot was cold and insensible, and the epidermis had begun to separate. The ankle-joint was exposed; the ligaments were destroyed, and the tendons, being no longer restrained, were extended like cords. The bones of the leg were also exposed in their lower third, and the tendons had begun to slough. Amputation was performed, and the patient recovered.

[The practice in this case was shameful; but the error was less in the employment of a particular bandage than in the subsequent inattention to the progress of the injured part. Any other bandage would probably have produced the same effects, when applied immediately after the accident, and suffered to remain for six weeks.] *Gazette Médicale. Juillet* 13, 1839.

---

### *On the Superiority of M. Reynaud's Operation for Circocele.* By M. Jules Roux, m.d.

M. Reynaud takes, with both his hands, the spermatic cord of the diseased side; he isolates and pushes inwards towards the root of the penis the vas deferens, whose hardness distinguishes it from the vessels and nerves of the testicle; pinching then the scrotum with the fore-finger and thumb of the left hand, so as to embrace the spermatic vessels and nerves, he passes through the base of the fold thus formed, a curved needle, with waxed thread. The scrotum then let go, there remains, between the entrance and exit of the needle, an interval of about an inch; upon this interval is placed a thick cylinder of lint, but not very long; and the two ends of the ligature brought together over it, and tied by a *bow-knot*, so as to admit of untying or relaxing, should it become necessary, to diminish its pressure. Small pledgets, with cerate, are put upon the punctures: no bandage is necessary to keep them, but a simple compress is placed over all. The patient remains in bed, with the scrotum on a cushion, and is treated with diluents and lavements. A few days after, inflammation round the ligature takes place, ordinarily slight, and not preventing a tighter tie of the contained parts. If, however, the inflammation be violent, or the pain excessive, M. R. unties and slackens the ligature, reduces the inflammation, and again reties it; which occupies about two or three days' time. As the soft parts become divided, the ligature is tied again with more pressure. Towards the fifteenth or eighteenth day, the nerves and vessels of the testicle have been divided, and there remains only the skin to be cut. M. Reynaud now passes a directory in the line of the thread, and divides the skin with a bistoury. A simple wound succeeds to this incision, and quickly cicatrizes; so that, in about twenty-five days from the commencement of the operation, the patient is ordinarily cured. Two cases have been successfully treated in this manner by M. Reynaud. [Does no mischief arise from compression of the nerve ?]

*Journ. des Connais. Méd.* No. v. *Feb.* 1839.

---

### *On Torsion of the Arteries.* By Dr. REMAK.

DR. REMAK recommends a modification of the operation of torsion of the arteries, which consists in seizing the vessel transversely with a pair of sharp wedge-shaped forceps, and then pressing forcibly, so as to divide the internal coat. The extremity of the arterity is then seized with another pair of forceps and twisted, while the torsion is prevented from extending up the artery by the first pair. The vessel is thus less injured than in the common proceeding, and the internal coat, which shrinks after being divided, offers an effectual barrier to the blood. The operation was tested experimentally upon a horse: the carotid was divided, and torsion, performed as recommended above, was sufficient to restrain the hemorrhage, even when the horse was made to trot briskly.

*Medicinische Zeitung.* No. vi. 1839.

---

### *On Congenital Dislocations of the Femur.* By M. BOUVIER.

AT the meeting of the Academy of Medicine, on the 16th of April, M. Bouvier presented three specimens of this rare malformation. One of them was from a woman, æt. 26, who had died of phthisis. Both femurs were dislocated. On the right side the head of the femur was still attached by a portion of the capsular ligament, which lay between the head and the outer surface of the ileum. The cotyloid cavities were reduced in size, and triangular; and, notwithstanding the wasting of the heads of the femur, the cavities were both too small to admit them.

The second case was one of double dislocation, in a woman, aged 29; but M. B. had received only the left hip-joint, and that, like those of the preceding subject, in an imperfect state. In the place of the cotyloid cavity there was only a slight superficial triangular depression, quite incapable of receiving the head of the femur, though it was much atrophied. The capsule was lengthened, and, as in the preceding case, interposed between the femur and the dorsum of the ileum. It was dilated at its extremities, but contracted, like an isthmus, at the middle, so that the head of the femur could not, during life, have been made to pass through it.

In the third case the left femur only was dislocated. The patient had been a porter, lame from birth, but robust. The head of the femur was higher and more forward than in the other cases. Enveloped above by its capsular ligament, it was placed on a small false articulating surface, on the ilium, to the edge of which the ligament was attached. The muscles attached to the upper part of the femur were all healthy, except the quadratus, which was atrophied, and in part fatty. On trying to draw down the head of the femur, by pulling it parallel to the axis of the body, and at the same time pushing up the ileum in the opposite direction, an insurmountable resistance was found to arise, from the tightness of the anterior and inner part of the capsule. But when the femur was strongly flexed and at the same time rotated very much outwards, and in this position (as in M. Desprez's mode of reduction) drawn parallel to the axis of its body, the head could easily be placed opposite to the acetabulum, though, from the contracted size and form of that cavity, it could not be placed in it.

[In reference to the important but still problematical question of the reducibility of congenital dislocations of the femur, these cases would show that the chief points to be considered are, the more or less complete obliteration of the cotyloid cavity, the contraction of the capsular ligament between the acetabulum and the part which surrounds the neck of the femur, the extreme resistance which the anterior and internal part of the capsule presents to any efforts at elongation of the limb, the difficulty of moving the thigh during life into the position in which, after death, a kind of reduction may be effected; and, lastly, the difficulty of retaining the limb during extension in the reduction which has been effected by its flexion.] *Bulletin de l'Acad. Roy. de Méd. Juin* 30, 1839.

---

*Chronic Hydrocephalus cured by Compression of the Head.*
By Dr. Löwenhardt, of Prenzlau.

Charlotte M., aged two years and a quarter, had enjoyed good health, till about nine months ago, when she received a fall on the head. Some weeks afterwards she was observed to be indisposed; she had become dull, her appetite had failed, and she was frequently affected with sickness, which, after a time, ended in almost constant vomiting.

Dr. Löwenhardt saw the child for the first time, about five months after the fall. He found her very much reduced, the skin shrivelled, and the face and feet œdematous. The head measured twenty-five inches, nine lines, in circumference, and the bones stood so wide apart, that Dr. L. could lay his fingers between the two frontal bones, at the upper part of the suture. There was no squinting, but the pupil was dilated and immovable; the respiration was unequal and sighing; the pulse slow and weak. The only nourishment which the child took was a little milk, which it almost immediately vomited; the bowels were relieved by injection. She did not seem to recognize even her mother, and she was completely lost.

In this apparently hopeless state, Dr. L. resolved to try the effect of compression, combined with other suitable remedies. The head was shaved, and three strips of adhesive plaster, which filled up the space between the eyebrows and the margin of the hair, were passed firmly round the head. A stream of cold water was poured from a height of two feet, for thirty seconds, four times a day, upon the vertex; a blister was applied to the nape of the neck, and kept open; frictions with mercurial ointment were made upon the neck and extremities; and, lastly, powders containing a quarter of a grain of calomel, combined with digitalis and chalk, were given twice a day.

In about a fortnight the vomiting had become less frequent, and the child now retained some milk upon the stomach. As it was found inconvenient to renew the adhesive plaster as frequently as was necessary, a strap with a buckle was substituted, which was occasionally drawn a hole tighter. The cold affusion was soon abandoned, from its wetting the strap, and the mercurials were given up as soon as the mouth showed symptoms of the system being affected, and diuretics were ordered instead.

The treatment had commenced on the 23d December, and on the 20th January the circumference of the head had diminished three-quarters of an inch. On the 27th February the circumference of the head was nineteen inches, seven lines; the bones were consolidated, and the appearance of the head was natural. The pupil had become moveable; the face, though pale, had a healthy appearance; the body had gained in size and strength, and the principal functions were in a normal state. Her gait, however, was staggering, and speech was very imperfect.

The child died of a species of typhus, about three years and a half afterwards. The body had continued to grow in strength, but the mental faculties had not kept pace with the corporeal development. *Casper's Wochenshrift.* No. 37, 1838.

---

*On the Treatment of Inguinal Hernia by Trusses.* By M. MALGAIGNE.

[WE give the following observations, not because we consider them applicable in their full extent to the surgery of this country, but because they contain remarks which will be very valuable to many, being founded on ample experience and well-digested and correct principles. The paper of M. Malgaigne we shall quote almost unaltered.]

The presence of a direct or oblique inguinal hernia shows a manifest predisposition to the development of a second; so that after an uncertain time, every one who has one hernia should reckon upon having another.

Every bandage hitherto devised for maintaining in place either a congenital or accidental oblique inguinal hernia is formed on a vicious principle, and requires a complete alteration. They all compress chiefly the external ring and act scarcely at all upon any part of the canal; the new principle which I wish to establish and which I have already applied, both in public and private practice, in a number of cases, consists in making pressure on the whole canal, but chiefly on the internal ring. The chief inconveniences of the old method are, 1, that, in closing simply the external ring, it allows the hernia to remain in the canal, and thus does no more than transform a complete into an interstitial hernia; 2, it only by chance produces a radical cure, and, even in children, the proportion of unsuccessful cases is enormous; 3, the hernia is evidently much less effectually detained, as is instantly confessed by the majority of those patients who have tried both methods; 4, when the hernia requires very forcible compression, all the bandages at present employed, by pressing on the pubes, compress the spermatic cord, and hence arises a frightful number of engorgements of the cord and of the testicle, an effect which is not produced by the new method. This method was applied and described by Sir A. Cooper, but is not known or is neglected in England, and is not mentioned in the writings of Samuel Cooper or Lawrence. This singular fact may possibly be somewhat explicable by the following consideration. Ruptures present themselves in practice under two general forms; either simple or reducible with facility, or complicated with serious accidents relating to their strangulation. The latter case, which is somewhat rare, requires prompt decision, and a dexterous and practised hand; it belongs to the domain of surgery, properly so called, and all the great and magnificent works of the modern schools have been chiefly engaged in the consideration of strangulated hernia. Surgeons have disdained the simple and reducible hernia; they have only superficially studied them, and they have abandoned their treatment to the hands of rupture-bandagers. So that these lesions, so numerous and so important, present in our days the strange anomaly, that surgeons study the disease, but do not occupy themselves about its treatment, and that bandagers are charged with the treatment of the disease without being acquainted with it. I was first struck with this state of things, when first appointed to the care of cases of hernia at the "bureau central" of the Parisian hospitals. The average number of those annually applying for trusses and pessaries is 3000; and in the two months of October and November 1835, I was able to collect 435 written observations, and to obtain results worthy of communication to two Academies. Since that time I have silently continued my work, wishing to arrive at results as complete as possible. During the last three years, my employment at the "bureau central" and in the hospitals, my connexion with the chief bandagers of Paris, and my private practice, have enabled me to see more than 2000 cases of hernia, to try almost every known bandage, and to determine the conditions under which bandages should be employed, and to place on exact foundations the science of prognosis and of indications. In this place, I merely wish to speak of the treatment of oblique inguinal hernia, the most common of all, and consequently the most important to the practitioner. The oblique inguinal hernia does not always present the same degree of development, and I have assigned to it the following degrees or periods: 1. When the hernia projects only through the abdominal ring; this I call *commencing hernia.* 2. When it occupies the inguinal canal; M. Goyrand has applied to this the name of *interstitial hernia,* a useful name to continue. 3. When it projects beyond the external ring; this is *bubonocele.* 4. When it descends into the scrotum; *oscheocele.* The latter two degrees are well known; the only practical difference which they present relates to the strangulation, which is more dangerous in bubonocele than in oscheocele, but in common cases they offer the same indications, and I shall not stop any further to notice them.

Interstitial hernia is often not recognized, unless it is very large, which is rarely the case; since, on applying the finger upon the external ring, no projection is felt; the complaints of the patient are ascribed to an imaginary feebleness of the abdominal parietes, or to some other cause. This degree of hernia is very common, and, as strangulation may happen in this case, it is necessary to fix to it serious attention. Lastly, *the commencing hernia,* the first degree of the disease, has been entirely neglected, both by bandagers and surgeons. The reasons of this are easily given. The patient never consults a surgeon at this period of his disease; and I confess that I have not yet had occasion to decide in a case of this kind. It is only in secondary hernia that I have learned to recognize it, after having appreciated all the importance of such a diagnosis; and as this importance results from a fact unknown before my time, it is not to be wondered at, that surgeons, occupied by a large hernia of one side, should be but little careful about an almost imperceptible swelling of the opposite side. But this swelling, however small it may be or may appear to be, is the certain sign of a near approach of a second hernia, and one may vainly check in the most perfect manner the primitive hernia, the second will not the less certainly appear as soon as this slight projection has been perceived. I will return to the subject of these secondary herniæ; I now wish only to establish the course which inguinal hernia generally follows in its development, as far as the fact bears upon treatment.

Hernia most frequently passes successively through these four degrees. Thus, an individual exerts himself and feels a crack in the inguinal region; he sees nothing at first, and it is only after eight or fifteen days that he sees a small tumour projecting beyond the ring, and afterwards the bubonocele becomes oscheocele. From this account, which is applicable to the majority of patients, one may conclude that the first effort has opened the internal ring, and that the hernia has subsequently passed to the extreme degree. Frequently, in these accidental herniæ, the intestines descend at once into the canal; I have seen some of them suddenly pass to the third degree; and sometimes the hernia becomes strangulated at the moment of its production. But I have never known an accidental hernia suddenly arrive in the scrotum. Each of the first three degrees may remain a longer or shorter period: thus, for example, I have seen the interstitial hernia develope itself so as to acquire half the size of the first, and remain for many years without escaping from the inguinal ring; sometimes, after having made its nidus in the canal, it finishes by making its escape externally; and this long delay in the canal, recognizable by the dilatation of its anterior wall, appears to me to be the essential cause, not hitherto recognized, of the displacement of the vessels of the spermatic cord. This being the state of things, one may judge of the advantage derivable from the application of bandages to the external ring, according to the common method. They transform bubonocele or oscheocele into interstitial hernia, and only prevent strangulation by the external ring, leaving the individual exposed to the danger of strangulation by the abdominal ring. They do not even remedy the common inconveniences of simple hernia; and as the pad does not allow anything to escape externally, and very much conceals the projection of the canal, the continuance of inconvenience has been attributed to the most fanciful causes. [A case is related showing the inutility of the ordinary bandages. And M. Malgaigne continues,] I might multiply cases of this kind; but any one may make upon the first hernia which he witnesses, an experiment which will lead to the same results. Apply the thumb upon the external ring and nothing escapes externally; the patient says that his hernia is kept up. Place the thumb upon the internal ring, and the patient will say that it is much more effectually kept up, and that he finds, on making any exertion, that there is greater firmness of the abdomen. One may easily understand, also, that as the hernia is not completely maintained by the common mode, a definitive cure cannot be obtained. The external ring only is acted on; the obliteration, if it take place, is only of the external ring, and the canal will always remain open for interstitial hernia. Now and then, as exceptional cases, cures have taken place; but as it has not been possible to reproduce them with any certainty, by the same means, the facts have been doubted. It has been simply believed that the truss sufficed for the cure of young subjects; nevertheless the number of exceptions to this would scarcely be believed. M. Bourat, one of our best bandagers, told me, with an air of triumph, that, out of fifty children, he probably did not fail to cure ten. But this I cannot, from my own experience, believe. I have had innumerable cases of congenital hernia under my own eyes, which have existed twenty, thirty, forty, and in one instance, fifty-three years, notwithstanding the employment of the common bandage. But how comes it that some have been cured, but that others have not? In my opinion, this depends on the form and on thesize of the pad. If the pad rest only on the external ring, there will be no more cases of cure in children than in adults. But if by a happy circumstance, the pad be badly made, too large for the object had in view, it rests on the canal, and may effect its obliteration. In young subjects, the canal is so short as to be readily compressed by a pad of moderate size, even badly placed; and the greater vitality explains the more frequent success; but in the adult, very large pads would be required to produce the same effect; and in simple cases, too large a pad is considered as ill made: I have seen a cure of this kind entirely accidental. Lastly, as a pad of moderate size may rest upon the external ring, without being at all supported by the os pubis, it happens, if the pressure is at all considerable, that pain and excoriations of the skin are the consequence, together with engorgements of the cord terminating in varicocele or engorgements of the testicles. Many patients, treated during late years by the wooden pads of Carpenter, which rest upon the external ring and the pubis, have been compelled to give them up in consequence of pains and excoriations. M. Devergie sent to me a young man with a double inguinal hernia, and at the same time, an orchitis of each side, the effect of pressure by a common truss. Excepting the complication of hernia with the testicle in the canal, I cannot imagine a more embarrassing case. I have examined at the "bureau central," in a certain number of individuals, what was the proportion of engorgements of the cord and of the testicle. In 200 cases of hernia, I have found sixty-five lesions of this kind; i.e.,

| | |
|---|---|
| Engorgements of the cord, most frequently with varicose dilatation | 40 |
| Engorgements of the testicle . . . . . | 23 |
| Atrophy of the testicle . . . . . | 1 |
| Hydrocele . . . . . . | 1 |

One must not, certainly, attribute all these secondary lesions to the action of the truss; for I have found varicocele and engorgements of the cord and of the testicle in individuals who have had old hernia, but who had never worn a truss; and here the cause is the pressure of the hernia itself. And it may be asked, if in the other cases the pressure of the hernia would not have sufficed to produce the same effect; but, in addition to the fact that the action of the truss is too direct to admit of denial, the most favorable conclusion for the common method would be that it is as likely to be the means of producing engorgements of the testicles as herniæ which are not reduced.

The motive which has induced M. Malgaigne to write this paper, is that the old method of employing trusses is almost universal in Paris, and because, as he adds, although the revolution is made in the science, it still requires to be made in practice.

The consequences deducible from what has been said, are, that every hernial bandage which presses upon the external ring, in inguinal hernia, is a bad bandage; and that the first principle applicable to the maintenance of hernia within the abdomen is to make pressure upon the internal ring and upon the canal. The form, dimensions, and degree of pressure of the pad are subjects to be hereafter treated of, together with the questions—In what cases is a radical cure, by means of a truss, possible? and what are the indications and chances of success?

*Bulletin gén. de Thérapeutique.* Tome xvi. 1839.

